# Unveiling the socio contextual triggers of smokeless tobacco use among blue collar workers: implications for workplace health and policy

**DOI:** 10.1038/s41598-025-24275-2

**Published:** 2025-11-18

**Authors:** Anupam Bandyopadhyay, Subhajit Pahari, Munmun Patra Pahari, Sarika Keswani

**Affiliations:** 1 GITAM School of Business- GITAM (deemed to be University), Bengaluru, India; 2 School of Business, Woxsen University, Hyderabad, India; 3 Symbiosis International (Deemed University), Pune, India; 4 Symbiosis Centre for Management Studies (SCMS Nagpur),, Nagpur, India

**Keywords:** Socio-contextual risks, Smokeless tobacco, Blue-collar workers, Occupational health, Structural equation model, Mediated moderation, Neuroscience, Psychology, Environmental social sciences

## Abstract

This study investigates the socio-contextual determinants of smokeless tobacco (SLT) use among blue-collar workers in India, a demography exhibiting the world’s highest SLT use. The Social Ecology Theory is used to understand the phenomenon by analysing the data collected from 392 blue-collar workers, through the mediated moderation approach in the structural equation model. The findings indicate that working conditions (*β* = 0.17; *p value* = 0.007), social norms and support *(β* = 0.14; *p value* = 0.011), personality (*β* = 0.09; *p value* = 0.014), and perceived effectiveness of SLT control policies (*β*= -0.13; *p value* = 0.002) significantly impact the SLT use of blue collar workers. However, knowledge about the health effects of SLT use and neighborhood disadvantages did not exhibit a significant influence. The theoretical modelling helped identify direct effects and underlying pathways through which environmental and psychological variables jointly shape tobacco behavior. Despite limitations inherent in self-reported data, this research provides actionable guidance for policymakers and organizations to develop targeted public health policies and workplace strategies to reduce SLT use among blue-collar workers.

**Supplementary Information:**

The online version contains supplementary material available at 10.1038/s41598-025-24275-2.

## Introduction

Tobacco consumption continues to be one of the most persistent global public health challenges of the 21 st century. While cigarette smoking often dominates the discussion, smokeless tobacco (SLT) has emerged as a particularly concerning form of tobacco use in low- and middle-income countries, where its consumption is both widespread and socially embedded. Unlike cigarettes, SLT products are often perceived as less harmful, more culturally acceptable, and even more convenient in occupational environments where smoking may be restricted. This perception, however, masks serious health risks. SLT use is strongly associated with oral cancers, periodontal disease, cardiovascular problems, and premature mortality, making it a critical but under-prioritized public health issue^1,2^. India stands at the epicenter of the global SLT crisis. With nearly 29% of adults reporting current use^3^, the country accounts for the highest number of SLT users worldwide. The ubiquity of products such as gutkha, khaini, and betel quid with tobacco reflects not only their affordability and accessibility but also their deep entrenchment in cultural practices and workplace routines. Among Indian workers, particularly those in blue-collar occupations, SLT use has become normalized as part of daily life- taken during breaks, shared among colleagues, or used to combat fatigue and hunger during long, strenuous shifts^4^. These patterns illustrate that SLT use is more than an individual habit; it is woven into the social and occupational fabric of working-class life.

Blue-collar workers are especially vulnerable to SLT use based on the contexts of their work and their underlying socioeconomic vulnerability. It is common for workers in this group to be exposed to long hours in physically troubling settings for low wages, without access to occupational health care. In these conditions, SLT use is normalised as a coping strategy for physical exhaustion, stress, and boredom. In fact, workplace cultures often normalise SLT use through peers and other social influences on communities that continue to operate with economic hardship and abiding demand for cheaper, available products^5^. In the end, blue collar workers are at risk of more disease and less productivity, but they are also likely experiencing debt because of their own tobacco use. The ramifications extend well beyond the individual since families are taking on care costs and employers are taking on the role of losing labour^6^. Socio-contextual factors including economic circumstances, occupational stress, and cultural acceptability formally sustain SLT consumption, based on these studies of using SLT in a discrepancy of the public’s ideas about how it is culturally acceptable^7^.

Yet, much of the tobacco literature has historically emphasized either the epidemiological prevalence of use or the clinical outcomes of disease, offering limited insight into how everyday social and occupational contexts shape behavior. Epidemiological data are indispensable, but they often fail to explain why, in certain groups, tobacco use persists despite awareness of health risks. For blue-collar workers in India, these questions are especially pressing because their consumption is not only a matter of individual choice but also a reflection of collective workplace culture, community norms, and structural disadvantage. Despite extensive epidemiological research on tobacco use, prior studies have rarely examined the socio-contextual triggers of smokeless tobacco (SLT) use in occupational settings, particularly among blue-collar workers in India. Most investigations have focused on individual-level factors or clinical outcomes^2,5^, overlooking the broader ecological influences such as workplace stressors, peer norms, and perceptions of policy effectiveness. Research in South Asian contexts has highlighted cultural acceptance of SLT^8^, yet the role of occupational stress, workplace culture, and social support in sustaining these habits remains underexplored^9,10^. Moreover, while Social Ecology Theory^11,12^ has been applied to smoking behavior, its integration with SLT use among Indian labor populations is limited. Finally, existing interventions often lack contextual sensitivity, leading to modest success in cessation outcomes^6^. This study addresses these gaps by applying Social Ecology Theory to model the multi-level determinants of SLT use among Indian blue-collar workers, using a structural equation modeling approach to identify both direct and indirect pathways through which socio-contextual and psychological variables shape tobacco behavior. So, the study aims to investigate how workplace conditions, social norms, neighborhood disadvantage, personality traits, health risk knowledge, and perceptions of SLT control policies collectively influence smokeless tobacco use among Indian blue-collar workers, using Social Ecology Theory and structural equation modeling.

In doing so, the study takes a holistic perspective when examining SLT use, since it is a behavior that is shaped by personal, social, and structural forces all at once. For instance, workers’ workplace conditions may include long shifts, significant physical strain with limited breaks, and absence of sleeping opportunities; in these situations, SLT may be a coping mechanism. The social norm aspect of SLT is enforced by peers and family, which often makes quitting difficult, and neighborhood disadvantages such as poor living conditions and recreational opportunities, limit access to healthier ways to cope. Personality also plays a role in the balance of individual, social, and structural context by providing individual contingencies by which people respond to stress and peer pressure. In addition to contexts, workers’ awareness of the health-related consequences of SLT and their beliefs (and how salient they are) about policies directed toward controlling SLT (e.g., bans or health warnings) will either trigger or weaken use (as a coping behavior). To confirm our theoretical assumptions and to capture the dynamic nature of SLT under a holistic perspective, the study collected data from 392 blue-collared workers’ responses to structured questionnaires that sought to measure workplace conditions, personality traits, peer and family’s social norms, neighborhood disadvantages, and perception of SLT control policies. Data will be analyzed using structural equation modeling (SEM), which provides measurement models and can simultaneously examine direct, indirect, and moderated relationships among variables. In particular, a mediated moderation approach is employed to explore how contextual factors (e.g., workplace conditions) interact with mediators such as social norms and health knowledge, while also considering the moderating role of policy perceptions. This approach provides a richer understanding of how SLT behavior emerges from the interaction of environmental, psychological, and policy-related variables. The research is guided by six core research questions:

### RQ1

How do working conditions influence SLT use among blue-collar workers?

### RQ2

What role do social norms and peer & family support play in SLT use?

### RQ3

How do neighborhood disadvantages affect SLT use behavior?

### RQ4

How do personality traits contribute to SLT use?

### RQ5

How does knowledge of health risks influence SLT use?

### RQ6

How do perceptions of SLT control policies shape use behavior?

These questions are important because they shift the dialogue about tobacco use away from an individualistic focus to highlight the larger ecological environment where consumption takes place. By situating the use of SLT within the realities of the worker’s world, this study advances its meaning - not only in terms of contribution to public health, but the consequences for economic productivity, stability in the household, and performance in the workplace. Illness related to SLT use can reduce income and exacerbate poverty, trapping marginalized populations in cycles of marginalization. Furthermore, workplaces that allow or condone the use of SLT are creating cultures that are harmful to the long term health of workers. In contributing to the field of tobacco control research, this study advances a multi-level understanding of SLT use through the application of Social Ecology Theory, emphasizing the interconnected influence of individual, social, and structural factors. . So the research links individual characteristics of workers, insightful work experiences, contextual circumstances that can be derived from the community and perceptions of health policy, to advance evidence for effective interventions. Moreover, evidence that expands the overall narrative contributes to useful insights for public policymakers, health clinicians, and workplaces interested in viable and effective practices. Conventional interventions often fail precisely because they overlook context; this study emphasizes that successful solutions must engage with the lived realities of workers and address the structural forces that sustain SLT use. The remainder of the paper is organized as follows: Section 2 outlines the theoretical framework, reviews relevant literature, and develops the hypotheses. Section 3 details the methodology used for data collection and analysis. Section 4 presents the results of hypothesis testing, and Section 5 discusses the implications for policy and practice, while also noting limitations and directions for future research.

## Theory, literature review, and hypothesis

### Social ecology theory (SET)

Social Ecology Theory (SET) was developed by Bronfenbrenner^11^, and adapted by McLeroy et al.^13^ for applications in public health. SET represents multiple systems at play that interact with each other as they relate to health behaviour (individual, interpersonal, organizational, community and policy). SET offers a useful model when investigating tobacco use and research. Some examples, such as, Sorensen et al.^12^, have demonstrated that systemic and environmental factors, along with the individual factors, can sustain unhealthy behaviours. Sorensen et al.^12,14^ used a social-contextual adaptation of SET that provides a contemporary assessment of the influence of social interactions within changes to tobacco use among blue-collar workers within the United States. The study found that tobacco use persistently occurred as a result of workplace norms, job stress, and the poor enforcement of a ban on smoking in the workplace. Sorensen et al.^15^ applied a similar social contextualisation when investigating tobacco control interventions among teachers in Bihar, India, to demonstrate how contextual influences combine to influence individual behaviour to use tobacco.

However, there are still debates when compared to other models regarding the sufficiency of SET. For instance, theories such as the Theory of Planned Behaviour (TPB) and the Theory of Reasoned Action (TRA) are becoming increasingly popular in explaining tobacco use behaviour and correlate using attitudes, subjective norms and behavioural intention^16,17^. These models provide valuable insights into individual-level decision-making, but they have been criticized for neglecting broader environmental and structural determinants that sustain addictive behaviors, especially in high-prevalence contexts like India^5^. While models such as the Theory of Planned Behaviour^16^ and the Theory of Reasoned Action^17^ emphasize individual intentions and attitudinal determinants, they offer limited scope to capture the broader workplace and policy-level influences that shape SLT use. In contrast, SET accommodates these complexities by explicitly incorporating multi-level socio-contextual factors—such as interpersonal support, occupational stress, neighborhood disadvantage, and policy enforcement—into a single framework. This breadth allows for a more comprehensive explanation of SLT use among blue-collar workers, where individual choice is inseparable from environmental pressures. By placing tobacco usage within intersecting systems SET expands the conceptual landscape of research and provides a more robust evidence base to inform interventions that target not only individual behaviors but also workplace culture and policies. Considering the complex nature of SLT use in Indian labor populations, SET provides the most fitting theoretical perspective for the current study.

### Literature on tobacco control

Findings from tobacco control research provide mixed evidence on the effectiveness of tobacco control interventions and existing evidence suggests that struggles exist in addressing tobacco use behaviors in low and middle income countries (LMIC). Public health awareness campaigns have increased tobacco related knowledge, however knowledge has not led to sustained behavior change in the context of cultural acceptance and social norm for tobacco use^5^. Public health experts support increasing taxation and pricing policies, and in some LMIC context cigarette consumption has decreased^18^, however SLT use remains prevalent as most products, are manufactured in the unorganised sector and sell at affordable prices^19^. Some authors note that governments must pass strong legislation and correspondingly enforce legislation such as bans on tobacco use in the workplaces or the creation of penalties for breaches, in combination with an awareness campaign, yet implementation was either weak or inconsistent^20,21^. There is also inconsistent evidence regarding the role of knowledge, while one study showed a relationship between knowledge of health risks with lesser initiation of use or more attempts to cease use^22^ and other authors indicate that knowledge was not a deterrent to use in a context where SLT was common is social contexts through peer pressure or workplace use practices^23,24^. These debates underscore the need for theoretical approaches, such as Social Ecology Theory, that move beyond individual-level predictors to account for the socio-contextual and policy environments in which SLT use persists.

### Hypothesis development

Adverse working conditions, which are a combination of the working environment and job characteristics, have been found to affect tobacco use positively^9^. According to the work-stress paradigm suggested in Frone & Bamberger^25^, job stress amongst workers results in physical and psychological health problems, thus encouraging risk-taking behaviours. Workers develop psychological issues and engage in health risk-taking behaviors in response to aversive working conditions^10^. These conditions include hazardous and toxic physical environments, heavy workloads, job insecurity, unfair pay, benefits, and promotions. The Demand-Control Model proposed by Robert Karasek [26] has been extensively used to establish a relationship between job stress and adverse health outcomes among workers^27,28^. Baek et al. and Chaudhary et al.^29,30^ found that several workplace factors increase the frequency of tobacco use. These factors include greater physical exhaustion, unhealthy environments, workplace boredom, role ambiguity and conflict, and inadequate resources. They also include underutilization of skills, delayed or insufficient pay, lack of fringe benefits, and unfriendly social and professional relations among employees. Evidence shows that psychosocial stress and peer influence drive health risk-taking behaviors. However, researchers have not determined whether these motives explain the widespread use of SLT among blue-collar workers in India. Hence, we develop the following hypothesis:

#### H1

 Working conditions have a significant positive impact on SLT use.

#### H1a

 Social norms and support positively mediate the relationship between working conditions and SLT use.

While adverse working conditions create stressors that may push workers toward SLT use, the persistence of such behaviour cannot be fully understood without considering the social environment that normalizes and sustains it. Social norms and support from the other members of society and family are critical aspects of tobacco use. For instance, parents’ tobacco use influences the initiation of tobacco use in adolescence. In adulthood, support from a spouse and other family members sustains the habit^31,32^. Any particular habit, even if it is unhealthy and practised by most society members, may appear normal and become an accepted social norm. SLT use is customary in many South Asian countries due to the large-scale prevalence of SLT use^8^. Certain social norms within a group may compel the members not to diverge from the perceived norms to avoid suffering social sanctions^33,34^. According to the Theory of Planned Behaviour, positive attitudes and subjective normative beliefs about smoking create the intention to smoke, resulting in smoking behaviour^16^. Also, the Theory of Reasoned Action (TRA) suggests that attitudes and beliefs about tobacco use and its social consequences predict smoking behaviour^17^. Moreover, the social learning approach observes that individuals are influenced by how common they consider smoking among the general population and how common it is among more desirable people, such as the successful and the elite^35^. Hence, it will be interesting to investigate the influence of social norms and social support on the behaviour of SLT use, since SLT use is a well-established social norm in the country. Thus, we develop the following hypothesis:

#### H2

 Social norms and support have a significant positive impact on SLT use.

Beyond workplaces and social circles, the broader neighborhood context also plays a powerful role in shaping health behaviors, including SLT use. The neighbourhood and the surroundings where an individual lives and grows up influence physical and mental health^36^. Lee et al.^37^ associate affluent, well-maintained neighborhoods with good health, whereas they find that deprived neighborhoods adversely affect residents’ lives. Neighbourhood disadvantage is an aggregation of adverse living conditions such as crime rates, lack of mutual trust, safety, health services, and education infrastructure related to the place of residence^38^. Ribeiro et al.^39^ show that disadvantaged neighborhoods harm physical health. These areas feature sub-standard housing, limited access to health services, and weak social organization. They contribute to stress-related diabetes, low physical activity, and poor weight management. Thus, the residents of disadvantaged neighbourhoods, in particular, socioeconomically underprivileged areas, are more apt to develop health risk-taking behaviours and supporting norms, such as tobacco use^40^. The study of the influence of neighbourhood disadvantage on the SLT use behaviour of manual labourers is crucial since they belong to the socioeconomically lower sections and are more likely to reside in deprived or disadvantaged neighbourhoods. Hence, we develop the following hypothesis:

#### H3

 Neighbourhood disadvantages have a significant positive impact on SLT use.

#### H3a

 Social norms and support positively mediate the relationship between neighbourhood disadvantages and SLT use.

Individual factors can mediate individual response to structural factors like workplace, familial and neighborhood factors. In this regard, knowledge of health risk is an important influence on whether an individual acts in response to structural factors and social pressures, or not. Knowledge of health risks associated with SLT is especially important in the initiation, maintenance, and cessation of SLT use. Studies show that individuals who understand the adverse health impacts of SLT- including oral cancer, heart disease, or reproductive health- are more likely to avoid or mitigate their use of SLT^22,23,41^ Festinger’s^42^ theory of cognitive dissonance helps to explain this finding; knowledge of health risks related to SLT can produce uncomfortable internal stimuli that prompts behavioral change if an individual continues to voice a willingness to experience risky behavior. What do we make of social contexts? In social contexts- like India- where risky consumption of SLT use is normative and widely accepted, awareness about SLT health risks may not manifest into behavioral change^5^. However, individuals who possess and are aware of health risks are more likely to respond to public health messaging and are willing to accept cessation support from health organizations^43^, all of which suggests knowledge is still a vital component for reduction in SLT use . Therefore, we can infer:

#### H4

 Knowledge about the health effects of SLT use negatively impacts SLT use.

Even with similar knowledge and environmental exposures, not all individuals respond in the same way; personality differences can help explain these variations in SLT use. Personality often explains the reason for deviations between two individuals under similar conditions. Thus, personality traits are the most stable and significant predictors of behaviour^44,45^. Similarly, in the case of health behaviours, personality has been found to influence the health habits of individuals^46^. As health behaviour is essentially the interaction of personal characteristics and the social environment, the evidence of personality as a predictor of tobacco use is abundantly available in the literature^47,48^. According to the Problem Behavior Theory, personality variables such as sensation seeking, impulsivity, and rebelliousness interact with social factors to predict tobacco use and other high-risk behaviours such as alcohol and substance abuse^49^. Similarly, the theory of triad influence posits that interpersonal, attitudinal, and intrapersonal characteristics are the three vital influences on an individual’s tobacco use behavior^50^. Tobacco use has also been found to be a coping mechanism against various kinds of psychosocial stress arising out of the social interactions of human existence^9^. Specifically, the Diathesis-Stress Theory postulates that the individual stress response depends on the personality (diathesis) traits of the individual, such as extraversion and introversion, which are the main distinguishing factors in handling stress^51^. Zou et al. and Farooqui et al.^52,53^ show that low self-esteem makes individuals vulnerable to depression. Depressive symptoms and stress create negative affect. This adverse affect increases the intensity of tobacco use. Thus, personality type plays a vital role in SLT use, and researchers must understand its impact in developing cessation interventions. Hence, we develop the following hypothesis:

#### H5

 Personality has a significant influence on SLT use.

**Fig. 1:**
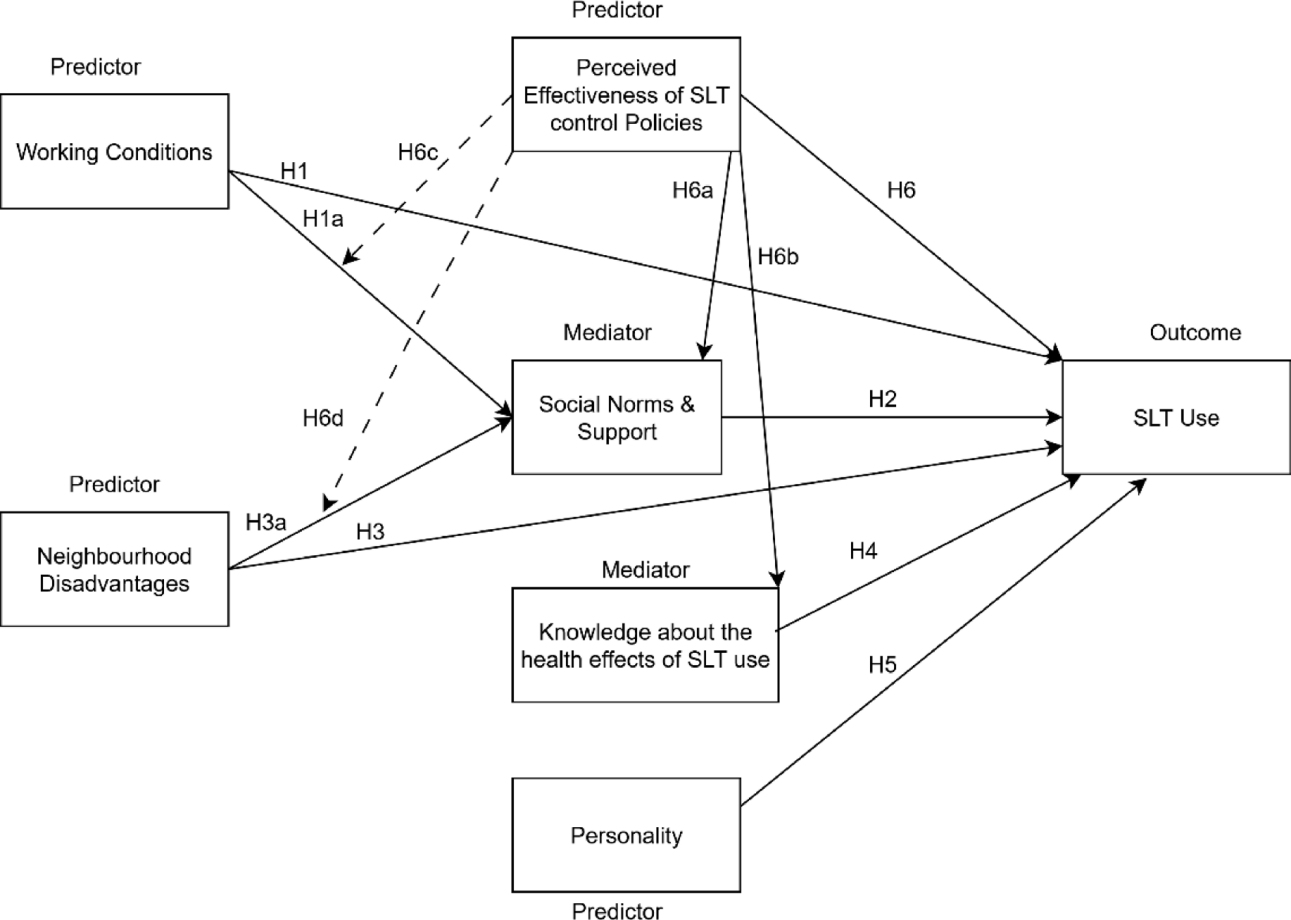
Conceptual framework and hypotheses.

Lastly, beyond individual and social influences, government tobacco control policies create the broader regulatory environment that can either reinforce or discourage SLT use. The government regularly implements several tobacco control policies to diminish the disastrous effects of tobacco use on public health^54^. The tobacco control policies are usually a combination of persuasion through awareness campaigns encouraging people to quit tobacco use and enforcing strict tobacco control legislation^20,55^. Through public awareness programmes, individuals are educated about the harmfulness of tobacco use, to bring a cessation of tobacco use among existing users and stop prospective users from developing the habit of tobacco use^43^. However, Siddiqui et al.^56^ suggest that creating awareness alone is insufficient to change risk-taking behavior, especially in quitting SLT addictions. Hence, policymakers must accompany public awareness campaigns with stringent legislation, such as restricting the sale of SLT products or reducing their supply. For instance, increasing taxes on tobacco products has been used as an effective measure of tobacco control worldwide^18^. However, in India, the demand for SLT products is catered mainly by the unorganised sector. Thus, the increased taxes do not often lead to a higher price, therefore failing to cause much of a deterrent to the users^57^. Hence, there has been a lot of emphasis on building awareness through anti-SLT campaigns, and such campaigns have been extensively visible in the mass media in recent years^58^. Subsequently, many testimonials were broadcast and aired on television and movie theatres, along with mandatory health warning messages about tobacco use, including SLT products. However, the effectiveness of these campaigns depended on how the intended beneficiaries - the existing SLT users- perceived the messages. Hence, we develop the following hypothesis (Fig. 1; Table 1):

#### H6

 The perceived effectiveness of the existing SLT control policies negatively impacts SLT use.

#### H6a

 Social norms and support mediate the relationship between perceived effectiveness of the existing SLT control policies and SLT use.

#### H6b

 Knowledge about the health effects of SLT use mediates the relationship between perceived effectiveness of the existing SLT control policies and SLT use.

#### H6c

 Perceived effectiveness of the existing SLT control policies moderates the indirect impact of working conditions on SLT use through social norms and support.

#### H6d

 Perceived effectiveness of the existing SLT control policies moderates the indirect impact of neighbourhood disadvantages on SLT use through social norms and support.

**Table 1:** Research questions and corresponding Hypotheses.

Research Questions	Hypotheses
**RQ1**: How do working conditions influence SLT use among blue-collar workers?	**H1**: Working conditions have a significant positive impact on SLT use.
	**H1a**: Social norms and support positively mediate the relationship between working conditions and SLT use.
**RQ2**: What role do social norms and support from peers and family play in SLT use?	**H2**: Social norms and support have a significant positive impact on SLT use.
**RQ3**: How do neighborhood disadvantages affect the SLT use behavior of workers?	**H3**: Neighbourhood disadvantages have a significant positive impact on SLT use.
	**H3a**: Social norms and support positively mediate the relationship between neighbourhood disadvantages and SLT use.
**RQ4**: How do personality traits contribute to SLT use among blue-collar workers?	**H4**: Knowledge about the health effects of SLT use negatively impacts SLT use.
**RQ5**: How does Knowledge about the health effects of SLT use affect SLT use behavior?	**H5**: Personality has a significant positive impact on SLT use.
**RQ6**: How do workers perceive existing SLT control policies, and how do these perceptions influence their SLT use?	**H6**: The perceived effectiveness of the existing SLT control policies negatively impacts SLT use.
	**H6a**: Social norms and support mediate the relationship between perceived effectiveness of the existing SLT control policies and SLT use.
	**H6b**: Knowledge about the health effects of SLT use mediates the relationship between perceived effectiveness of the existing SLT control policies and SLT use.
	**H6c**: Perceived effectiveness of the existing SLT control policies moderates the indirect impact of working conditions on SLT use through social norms and support.
	**H6d**: Perceived effectiveness of the existing SLT control policies moderates the indirect impact of neighbourhood disadvantages on SLT use through social norms and support.

v

## Methodology

The blue-collar workers, who had the daily habit of using SLT products, were selected mainly from industries involving heavy physical labour, such as manufacturing, mining, and construction. Amarasinghe et al.^59^ and Boal et al.^60^ find that SLT use is common in these industries because smoking is prohibited and holding cigarettes is impossible since workers’ hands are extensively occupied. The data was collected from the neighbouring states of West Bengal, Jharkhand, and Bihar in eastern India from January to June 2024. These states had a high prevalence of tobacco use, greater than the national average^3^.

Physical interviews with a structured questionnaire of respondents were conducted at their work site or the canteens during recess or breaks. The worksites were appropriately selected for interviews, since the workplace provided a unique opportunity to observe SLT use concerning the physical environment and job stress. Moreover, conducting interviews at worksites provided access to the workers who had come to work from the surrounding rural areas, who may have been difficult to reach. After explaining the questions to the respondents, the interviewer mainly noted the responses. The study adopted this method to reduce response bias because most respondents were poorly educated and had limited cognitive ability to comprehend the questions correctly. Researchers collected responses by stimulating discussion with the participants and making the questions lucid. This approach reduced non-response bias more effectively than a questionnaire-based method. The researchers translated the questionnaire into Hindi and Bengali, the dominant languages of the region that respondents understood well. Google Translate was used initially, and then to reconcile any differences in meaning, two bilingual experts, one from Hindi and another from Bengali, compared the translations and suggested improvements. Then, the questionnaire was back-translated into English to recheck the accuracy of the translation^61^. The survey instrument was pre-tested among a small sample of daily SLT users to identify the relevant items and discard the irrelevant items in the Indian context, discover the best interview method, and get an idea about the total time required for completing each questionnaire. Accordingly, the researchers modified the questionnaire, and the revised version took less time.

The questionnaire comprised three main sections- the first section collected information about the demographic characteristics; the second section had the modified version of Fagerström Test for Nicotine Dependence – Smokeless Tobacco (FTND-ST) scale to measure the intensity of SLT use; then final section collected information about the various explanatory variables of interest such as personality, neighbourhood disadvantages, social norms, knowledge of the health effects of SLT use, working conditions and the perceived effectiveness of SLT control policies. The study selected the questionnaire items mainly through a comprehensive review of the existing literature. Items were excluded because they were irrelevant in the Indian context or had become redundant. All the items in the questionnaire were positive statements to which respondents had to record their degree of agreement or disagreement on a five-point Likert scale. The study adopted a convenience sampling procedure for employing a survey interview method to collect the responses in a face-to-face meeting. The researchers obtained infomed consent from respondents before the interviews. They allowed participants to withdraw at any time and to skip any question. In total, they conducted 500 interviews. 392 Participants (71.27%) fully completed the questionnaires (Table 2).

**Table 2:** Characteristics of the respondents.

Variable	Frequency (*N* = 392)	Percentage (%)	Variable	Frequency (*N* = 392)	Percentage (%)
**Age** 15–24 years	14	3.6%	**Occupational Status** Permanent Employee	142	36.2%
25–34 years	71	18.1%	Contractual Employee	146	37.2%
35–44 years	101	25.8%	Self-employed	104	26.5%
45–54 years	128	32.7%			
55–64 years	76	19.4%	**Marital Status** **Married**	**312**	**79.6%**
65+	2	0.5	**Divorced/Separated**	**2**	**0.5%**
**Education Level** Illiterate	79	20.2%	**Widowed**	**7**	**1.8%**
Primary	123	31.4%	**Single**	**71**	**18.1%**
Secondary	79	20.2%	SLT use		
Higher Secondary	40	10.2%	Low	141	36
Graduation	58	14.8%	High	251	64
Post-Graduation	13	3.3%			

The outcome variable in the present study was SLT use (SLTuse), which is the physical dependence on nicotine, the main component in tobacco, causing dependence. The Fagerström Test for Nicotine Dependence – Smokeless Tobacco (FTND-ST) has been used to measure the physical dependence on nicotine of SLT users^61^. Researchers have found that the FTND-ST scale correlates significantly with serum cotinine among users with high nicotine exposure. Hence, the study found the FTND-ST scale suitable for use because it measures nicotine dependence in high SLT users. The FTND-ST was developed using the Fagerström Test for Nicotine Dependence^63^ and the Fagerström Tolerance Questionnaire^64^. FTND-ST has been widely adopted in clinical use and research for measuring nicotine dependence’s physiologic and behavioural parameters^65,66^. FTND-ST contains six items; the total score is the sum of the individual items, with ten as the highest possible score. The overall score obtained was converted into a dichotomous variable, having two categories with scores 0 to 5 (Low = 0) and 6 to 10 (High = 1).

The study used the shortened version of the Eysenck Personality Questionnaire-Revised (EPQR) to measure the Personality (PR) of the respondents^67^. In his biologically based personality model, Eysenck^68^ found that the psychometric properties and substance use correlated. Eysenck’s theory has both theoretical and practical value, as it emphasises the biological mechanism that triggers personality traits, particularly responsible for health risk-taking and deviant behaviours such as substance and tobacco use. Eysenck’s theory suggests that the nicotine in tobacco adjusts the cortical spur to attain an ideal state of excitement among regular tobacco users. For instance, regular tobacco users are strongly motivated by anxiety and anger to use tobacco, as nicotine reduces the negative affect and enhances positive feelings. Eysenck had conceptualized personality in three biologically based traits, namely extraversion, neuroticism, and psychoticism, and suggested that extraverts are usually under-aroused and bored. Hence, extraversion individuals need external stimulus in the form of nicotine from tobacco for an optimal level of performance. Similarly, neuroticism in an individual is characterized by a high level of negative affect, such as depression and anxiety. The neurotic individuals have low stimulation thresholds, experience negative affect in the face of moderately trivial stressors, and get easily distressed in adverse circumstances. The psychoticism in individuals depicts toughmindedness, non-conformity, hostility, and impulsivity, and such individuals tend to disregard common sense and behave impulsively. Past studies have extensively employed EPQR to measure the personality traits of SLT users and other tobacco users^69,70^. The other explanatory variables were *Neighbourhood Disadvantages (ND)*, *Social Norms and Support (SNS)*, *Working Conditions* (*WC*), *Knowledge about the health effects of SLT use (KHE)*, and *Perceived Effectiveness of SLT control Policies* (*PEP)*. The responses for these variables were recorded on a five-point Likert-type scale ranging from strongly disagree (1) to strongly agree (5) (Tables 3 and 4).

**Table 3:** The Fagerström test for nicotine Dependence-Smokeless tobacco (FTND-ST)-Modified.

Items	Options/Responses	Points
How soon after you wake up to do you place your first dip?	Within 5 min 6–30 min 31–60 min After 60 min	3 2 1 0
How often do you intentionally swallow tobacco juice?	Always Sometimes Never	2 1 0
Which chew would you hate to give up most?	The first one in the morning Any other	1 0
Original item : How many cans/pouches per week do you use? Modified item: On an average how many times do you take SLT/day?	More than 3 2–3 1 More than 15 10–15 1–9	2 1 0 2 1 0
Do you chew more frequently during the first hours after awakening than during the rest of the day?	Yes No	1 0
Do you chew if you are so ill that you are in bed most of the day?	Yes No	1 0

**Table 4:** Latent constructs, observed items and their source of adoption.

Constructs	Instrument Items	Reference
Personality (PR)	My mood often goes up and down I am a talkative person I would be worried if I had a debt I am a lively person I prefer to go my own way rather than act by the rules I am a nervous person I worry a lot when there is any problem I usually keep quiet on social occasions. I am worried if I know there are mistakes in my work. I am mostly quiet when I am with other people I often feel lonely It is better to follow society’s rules than go your own way	Eyesenck Personality Questionnaire- Revised short form (EPOR-S) (Eyesenck and Eyesenck, 1992); (Tiwari, Singh & Singh, 2009)
Neighbourhood Disadvantage (ND)	I trust most people in my local area My local area has reputation as a safe place In my local area neighbors look after each other I am better off in my local area compared to others	Siapush et al., 2006
Social Norm & Support (SNS)	Most of the people in my society use SLT Wealthy people use SLT more It is better to use SLT than to smoke or drink alcohol Visitors freely use SLT in my home My SLT use has increased due to the practice at my workplace Many of my close friends and family members use SLT SLT is more used when I am with others in a group I started using SLT after seeing my parents using it at home Sharing SLT helps in making friends with people	Adkinson et al., 2015 ITC-TCP- India (wave 2) Biener et al., 2010 Stuber, Galea & Link, 2008 Biener et al., 2010
Knowledge about the health effects of SLT use (KHE)	I know using SLT cause cancer (mouth, throat, etc.) I often think about the harm SLT use might be doing to me Nicotine is the main substance in smokeless tobacco that makes people use it I know using SLT cause diseases of heart	ITC-TCP- India (wave 2)
Working Conditions (WC)	I have to fully focus my attention during work I have to do/decide things where mistakes could be quite costly I have to talk with peers, supervisor and other staffs for my work My work is physically difficult My job interferes with my family life The physical surroundings of my workplace are pleasant I can usually decide when to work fast and when to take it easy My opinion is asked when decisions are made about my own jobs or about general problems in the work place My work helps me to learn and develop new and special skills The work I do is very interesting	Kawada & Otsuka, 2010 Albertsen et al., 2003; House et al., 1979 Hu & Cheng, 2010 House et al., 1979; Albersten et al., 2003 House et al., 1979 House et al., 1979 House et al., 1979; Kawada & Otsuka, 2010 Kawada & Otsuka, 2010 Hu & Cheng, 2010 House et al., 1979
Perceived Effectiveness of SLT control Policies (PEP)	Anti-SLT advertising is effective for diminishing its use There should be strong anti-SLT policies at the workplace The pictorial warnings on SLT packages are realistic The anti- tobacco advertising on television and movie theaters has made me more likely to quit using SLT	Raut & Pawar, 2016 ITC-TCP- India (wave 2)

The study conducted data analyses in several sequential stages. In the first stage, descriptive statistics were calculated to examine the distributional properties of the variables, outliers, and correlations among the variables. An Exploratory Factor Analysis (EFA) was done to reduce many variables into fewer factors or constructs. Also, in the context of scale adaptation, EFA helps determine if the original factor structure of the scale remains consistent across different cultural contexts. When applied to a new population, the scale’s structure may change significantly; it may not measure the same constructs, leading to inaccurate results^71^. Structural equation modelling (SEM) was used to simultaneously assess the associations between observed and latent variables (the measurement model) and the relationships amongst the latent variables (the structural model). The study followed a two-step process. First, Confirmatory Factor Analysis (CFA) tested the measurement part of the model. Then, they tested the hypothesized structural model. Based on the formulated hypotheses, they conducted analyses to identify significant structural paths between each construct and the outcome variable. Therefore, the direct and indirect effects of six socio-contextual variables (*PR*,* WC*,* ND*,* SNS*,* KHE*, and *PEP*) on the *SLTuse* were estimated while controlling for occupations and the level of education. When the moderated effect of two predictor variables (let us say, *WC* and *PEP*) is found to be mediated by another variable (let us say, *KA*), mediated moderation is supposed to take place^72^. The fitness of the model was tested using the model fit indices such as Root Mean Square of Error Approximation (RMSEA) and Goodness of Fit Index (GFI), Comparative fit index (CFI), Normed Fit Index (NFI) and χ^2^/degrees of freedom (χ^2^/df).

## Results

Data with a normal distribution is one of the prerequisites for estimating SEM. The skewness and kurtosis of individual measured items lay between ± 1 and ± 3, indicating that the data were close to normally distributed with little deviation from the mean of a random variable. The extraction of the factors through principal component analysis and varimax rotation found that six factors explained more than 70% of the variance, with Eigenvalues of more than 1 (*social norms and support*, *working conditions*,* personality*,* neighbourhood disadvantages*,* perceived effectiveness of SLT control policies*, and *knowledge about the health effects of SLT use (KHE)*. The constructs showed good reliability as their internal consistency values (Cronbach’s alpha) were above 0.70 (Table 5).

The extracted dimensions from EFA were used to construct the measurement model, and covariances were drawn between the constructs. The measurement model fitted the sample data well (χ^2^/df = 1.36; RMSEA = 0.03; NFI = 0.928; CFI = 0.980, and GFI = 0.902). The composite reliability (CR) and average variance extracted (AVE) were above the threshold (CR > 0.70; AVE > 0.5) values, and hence, the internal consistency and the convergent validity of the constructs were established (Table 3). The CR is used to test the scale’s reliability and primarily measures internal consistency, indicating how well the items in a scale consistently measure the same underlying construct. On the other hand, AVE measures convergent validity, indicating the extent to which a construct explains the variance in its indicators. To simplify, CR focuses on the relationships between the items, while AVE focuses on the relationship between the construct and its indicators or the items used to measure it. The multicollinearity problem was nonexistent as the Variance Inflation Factor (VIF) was less than the recommended value of 4^73^ (Table 6).

**Table 5:** Factor Loading, average of variance extracted (AVE), composite reliability (CR), Cronbach alpha (CA).

Construct	Items	Factor Loadings						α	CR	AVE
		1	2	3	4	5	6			
Social Norms and Support (SNS)	(SNS1)	0.92						0.97	0.85	0.58
	(SNS2)	0.92								
	(SNS3)	0.91								
	(SNS4)	0.90								
	(SNS5)	0.89								
	(SNS6)	0.87								
	(SNS7)	0.86								
	(SNS8)	0.86								
	(SNS9)	0.83								
Working Conditions (WC)	(WC1)		0.81					0.92	0.97	0.76
	(WC2)		0.78							
	(WC3)		0.78							
	(WC4)		0.78							
	(WC5)		0.76							
	(WC6)		0.76							
	(WC7)		0.75							
	(WC8)		0.75							
	(WC9)		0.73							
	(WC10)		0.70							
Personality (PR)	(PR1)			0.91				0.91	0.92	0.53
	(PR2)			0.91						
	(PR3)			0.85						
	(PR4)			0.84						
	(PR5)			0.76						
	(PR6)			0.70						
Neighbourhood Disadvantages (ND)	(ND1)				0.90			0.90	0.91	0.63
	(ND2)				0.88					
	(ND3)				0.88					
	(ND4)				0.83					
Perceived Effectiveness of SLT Control Policies (PEP)	(PEP1)					0.82		0.86	0.91	0.70
	(PEP2)					0.81				
	(PEP3)					0.80				
	(PEP4)					0.78				
Knowledge about the health effects of SLT use (KHE)	(KHE1)						0.81	0.83	0.83	0.55
	(KHE2)						0.81			
	(KHE3)						0.80			
	(KHE4)						0.68			

α = Cronbach Alpha (> 0.7); CR = Composite Reliability (> 0.7);.

AVE = Average Variance Extracted (> 0.5).

**Table 6:** Results of multicollinearity.

Variables	SLTuse	ND	PR	PEP	KHE	SNS	WC	VIF
SLTuse	1	0.07	0.08	− 0.17	−0.12	0.15	0.07	—
ND	0.07	1	−0.14	0.08	0.14	−0.03	0.05	1.041
PR	0.08	−0.14	1	−0.03	−0.05	−0.08	0.03	1.029
PEP	−0.17	0.08	−0.03	1	0.48	−0.13	0.13	1.317
KHE	−0.12	0.14	−0.05	0.48	1	0.09	0.14	1.325
SNS	0.15	−0.03	−0.08	−0.13	−0.09	1	−0.17	1.051
WC	0.07	0.05	0.03	0.13	0.14	−0.17	1	1.053

**Fig. 2:**
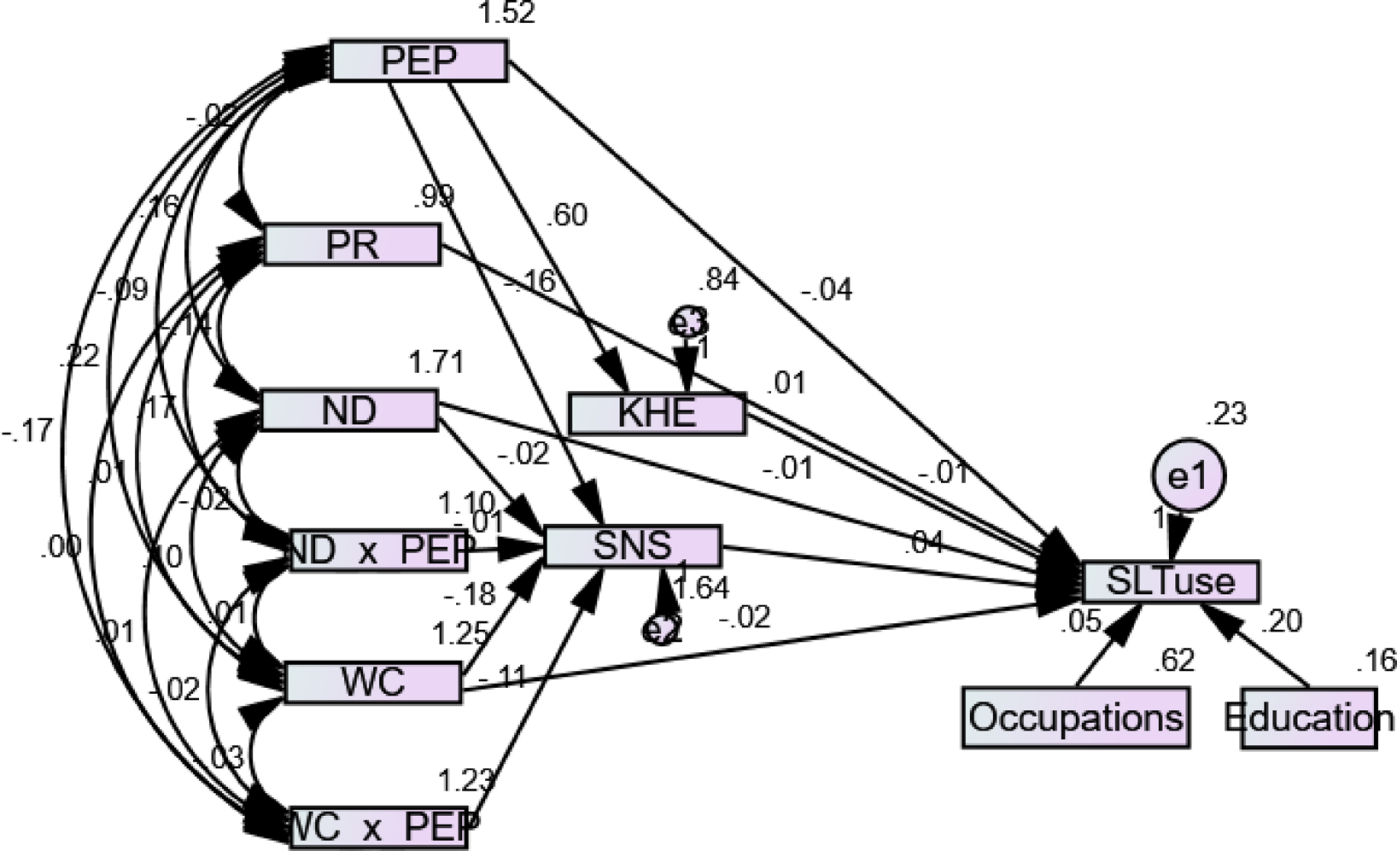
Amos output for Structural Equation Model (Model 1).

The structural model estimated all the hypothesised relationships between the latent variables. The structural model treated occupations and educational level as confounding variables. The researchers explicitly modeled these as observed variables within the SEM model (Fig. 2). They then estimated their direct paths to the dependent variables to statistically control their effects. This approach helped isolate the unique impact of the independent variables on outcomes. The analysis tested the model for the direct and indirect effects to test the significant paths from the predictor variable to the outcome variable. In keeping with the theoretical model, specific moderated impacts, with the interacting variables *PEP X ND* and *PEP X WC*, were mediated through *SNS;* thus, a mediated moderation occurred. The structural model was found to have an excellent fit (χ^2^/df = 1.58; RMSEA = 0.04; NFI = 0.958; CFI = 0.981 and GFI = 0.995).

**Table 7:** Direct and indirect effects of the explanatory variables.

Paths	Standardised β	*p*-value
**H1**: WC → SLTuse	0.05	0.367
**H1a**: WC → SNS → SLTuse	0.17	**0.007**
**H2**: SNS → SLTuse	0.14	**0.011**
**H3**: ND → SLTuse	0.03	0.533
**H3a**: ND → SNS *→* SLTuse	0.12	0.703
**H4**: KHE → SLTuse	−0.02	0.149
**H5**: PR → SLTuse	0.09	**0.014**
**H6**: PEP → SLTuse	−0.13	**0.002**
**H6a**: PEP → SNS → SLTuse	0.20	**0.017**
**H6b**: PEP → KHE → SLTuse	0.43	0.738
**H6c**: PEP × WC → SNS → SLTuse	0.13	0.328
**H6d**: PEP × ND → SNS → SLTuse	0.20	0.672

Table 7 presents the results of the significance of the various hypothesized paths. It is found that *WC* did not impact *SLTuse* significantly (*p value* = 0.367; *H1* rejected). However, a significant impact of *WC* was found on *SLTuse* when mediated through *SNS (β* = 0.17; *p value* = 0.007; *H1a* accepted). *SNS* was also found to significantly impact the *SLTuse (β* = 0.14; *p value* = 0.011; *H2* accepted). *ND* was also found not to impact *SLTuse* directly (p value = 0.533; *H3* rejected) or mediated through *SNS* (*p value* = 0.703; *H3a* rejected). Similarly, *KHE* did not significantly impact *SLTuse* (*p value* = 0.149; *H4* rejected). However, *PR (β* = 0.09; *p value* = 0.014) and *PEP* (*β*= −0.13; *p value* = 0.002) are found to have a direct significant effect on *SLTuse*. Thus, hypotheses *H5* and *H6* are both accepted. Additionally, *PEP* significantly impacted *SLTuse* mediated through *SNS* (*β* = 0.2; *p value* = 0.017; *H6a* accepted). However, *PEP* did not significantly impact *SLTuse* mediated through *KHE* (*p value* = 0.738; *H6b* rejected). The mediated moderation effects, when tested with the interacting variables *PEP X WC* and *PEP X ND*, through *SNS*, were not found to have a significant impact on the *SLTuse* when mediated through *SNS*. Thus, hypotheses *H6c* and *H6d* are both rejected.

### Confirmation of ethics approval

All experimental protocols were approved by the Ethics Committee of the author’s institution i.e. Symboisis centre for Management studies constituent of Symbiosis International Deemed university Nagpur, and all methods were performed per the relevant guidelines and regulations.

## Discussion

The findings of this study reinforce the view that smokeless tobacco (SLT) use among Indian blue-collar workers cannot be reduced to an individual lifestyle choice. Instead, it reflects the interaction of multiple influences operating across personal, social, and structural domains. By applying Social Ecology Theory^11,12^, the study highlights how factors at different ecological levels combine to sustain SLT use behavior, while also showing the indirect pathways through which these influences operate. A central result of the analysis was the significance of workplace conditions. Workers experiencing long hours, high physical strain, and limited rest were more likely to report SLT use. This aligns with previous research describing tobacco as a coping mechanism for occupational stress and fatigue^5^. Within the ecological framework, the workplace functions as a microsystem that exerts a direct influence on daily behavior. Yet the data also suggest that workplace stress alone is insufficient to explain consumption; its impact is amplified when social norms within the workplace normalize SLT use. This finding points to the importance of workplace culture, where the act of sharing SLT during breaks or shifts reinforces both social cohesion and consumption.

Social norms, both at work and in family life, emerged as strong determinants of behavior. Workers with peers or family members that accepted or promoted SLT were much more likely to maintain use, consistent with studies that have documented the cultural embededness of SLT use in South Asia^8^. Conversely, workers who reported discouragement from peers or family members did use less, indicating that social connections are both risk and protective factors. This duality is also an important realization of ecological thinking: social connections that might be hazardous can also be called into service for promoting better practices. These “ecosystems” were not entirely responsible for every SLT use at work, however. Neighborhood disadvantage added another layer of influence. Workers living in disadvantaged neighborhoods- whether empty calories and high populations, bad infrastructure, or lacking resources that promote health- reported much higher SLT use. These results build upon previous research documenting the effects of neighborhood disadvantage on substance use issues^9^, by demonstrating how neighborhood contexts may be shaping occupational health risks indirectly in other communities. From an ecological perspective, the neighborhood represents an exosystem on the individual, based on the stressor exposure or lack of access to healthier coping options.

Personality traits played a role, but their effects were in complex, interactive situations, rather than isolated traits. Impulsivity and risk-taking traits were associated with increased use of SLT, while self-control and conscientiousness correspondingly reduced risk. Further, these traits moderated the influence of the context as environmental stressors: workers who exhibited high characteristics of impulsivity, for instance, were more likely to respond to workplace stress through SLT use than workers demonstrating higher self-regulation. This suggests the application of ecological principle whereby individual-level dispositions interact with contextual penality to produce different behaviors through the interplay between person and environment. Health knowledge among many workers did not determine adverse health behaviors, for instance, workers who continued to use SLT even after knowledge of the negative impact did because workplace conditions and peer expectations presented a more immediate pressure. While knowledge remains an individual influence when considering an ecological model, when the social (interpersonal) and structural (environmental) forces are in play, the knowledge played little role in discouraging SLT use.

However, policy perceptions added an important aspect to the analysis. Workers who perceived that SLT control policies are credible and provable are less likely to use SLT, suggesting that perceived legitimacy and effectivenes are influencing compliance. This is consistent with previously established evidence that regulatory interventions have impact not always because of administrative enforcement capacity, but often because there is public trust that the policies enacted are meaningful and fair^10^. These perceptions connect to the macrosystem in the ecological perspective, demonstrating that health behaviors can be influenced by political and cultural systems. Thus, these findings reflect the utility of the Social Ecology Theory because they can explain the complicated relationship to SLT use among blue-collar Indian workers, as the theory describes several layers where behaviors emerge through the interaction of a number of levels of influence: individual (personality and knowledge); microsystem (familial or workplace norms); exosystem (neighborhood disadvantage); macrosystem (policies and cultural acceptance). Although the study did not connect these levels sequentially, it illustrates that SLT use is supported not by only one factor, but rather, the simultaneous convergence of stress, culture, structural disadvantage, and poor policy administration.

The indirect effects in this analysis are equally useful. For example, workplace stress predicted SLT use more strongly when mediated by peer norms; the effects of personality traits were enhanced or reduced depending on perceptions of policy effectiveness. These findings illustrate the concept of mediated moderation, where broader contextual factors shape behavior via mediating variables. This type of account emphasizes a more fluid and realistic account of tobacco use, which jives better with the messy complexity of people acting in ecological systems. To be clear, the study illustrates that SLT behaviors in the context of blue-collar work have to be understood by more than individual-level interventions. SLT behaviors result from a complex interrelationship of influences situated between daily workplace habits and national policy. By situating SLT behavior within this ecological perspective, the study contributes to a more holistic understanding of tobacco use in vulnerable labor populations and offers a foundation for developing multi-level, context-sensitive strategies.

## Implications

This study provides insightful implications for organisations and policymakers by presenting actionable recommendations for targeted interventions. Workplace tobacco cessation programs should extend beyond basic health education and incorporate stress management strategies, behavioral interventions, and peer-driven initiatives. Given the strong influence of social norms, these programs must focus on reshaping workplace culture by discouraging SLT use and promoting healthier alternatives. Employers can proactively implement SLT-free workplace policies, provide counseling services, and integrate cessation support programs tailored to workers’ needs. Highlighting both the economic and health benefits of quitting, in addition to helping ensure access to important resources (such as nicotine replacement therapy or professional counseling), may facilitate program effectiveness. A potentially very successful smokeless tobacco cessation strategy for blue-collar employees- would incorporate the peer mentor experience of a successful quitter. The mentor can experience success since it is usually recognized that quitting is oftentimes a personal, autonomous decision- an exercise of mental toughness rather than yielding to authority. Consequently, people particularly high on extraversion and neuroticism should be more heavily regarded during SLT quitting interventions. Habit substitution strategies, for example, chewing gum and nicotine replacement substitutes, utilizing short, high-intensity exercise, serve as both oral stimulation and self-efficacy while craving. Organizations can promote group check-ins as part team-building exercises that strengthen social ties & reinforce chosen norms related to health, work performance, and tobacco-free living. There are several example of tobacco cessation interventions in the workplace^74–76^. The academic research almost always finds tobacco cessation interventions mixed success rates. Directly contributing positively to motivation was financial incentives, management encouragement, and multiple sessions of counseling.

While peer pressure, work-related stress, and an inability to quit without medication were important barriers, public health campaigns need to transition from general awareness campaigns to efforts aimed at changing social norms related to SLT use. From a policy and regulatory perspective, SLT control policies need to be firmly enforced. Raising taxes on SLT products, limiting access to SLT products in workplaces, and legally enforcing restrictions on their sale will limit access to SLT. There also needs to be more collaboration between employers, policymakers, and healthcare providers to devise sustainable, context-specific interventions to address the sociocultural context of SLT use. Overall, they will contribute to the reduction of SLT use and improve the health and well-being of blue-collar workers and workplace productivity for employers while limiting the healthcare burden. The study also contributes to theoretical knowledge of socio-contextual influences on different tobacco consumption modalities in blue-collar workers. Specifically, the study adds a meaningful theoretical contribution to understanding SLT use in India by extending the Social Ecology Theory to better understand SLT use behaviours. There are limited tobacco control literature representations of this population. In addition, the topical study positively adds value by recognizing multi-level socio-contextual factors, to further the researcher’s understanding of tobacco use by including workplace setting, personality, social norms, and consequences of understanding policy; an opportunity where researchers typically have approached tobacco use patterns either individual- or clinical-level. Furthermore, utilizing a mediated moderation approach to modeling with structural equation modeling , the current research addresses both direct impacts and mediated moderation effects to explain the interrelatedness between environmental and psychological behavior factors. By capturing these perspectives, the current study has filled an important methodological and contextual gap by capturing the pathways for how SLT use persists in labour-market scenarios, particularly in low- and middle-income countries.

## Conclusion and limitations

To conclude, the study highlights the significant influence of workplace conditions, social norms, personality traits, and policy perceptions in sustaining SLT use, while factors such as knowledge and neighbourhood disadvantages exhibit limited influence. However, the present study has certain critical limitations that future research may address. First, convenience sampling and selecting a specific geographical location suffer from serious bias.

Convenience sampling includes using participants that were easily accessible and may not represent the rest of the target population. This generates a larger representation of some groups and less or no representation of others, thus narrowing generalizability of the study. In addition to the sampling not being random, the study participants may be different than the population in an unknown or uncontrollable way, potentially affecting internal and external validity. Convenience sampling may also yield smaller and less diverse samples that produced a lower statistical power, giving the study less chance to demonstrate real effects. Future studies may expand recruitment to include additional states, and include a greater geographical and culturally diverse area, while using probability sampling strategies, such as stratified or random sampling, to increase appropriate representation. Second, as we utilized self-report surveys, they may be subject to social desirability and recall bias. Hence, using more observational and objective forms of data collection during the survey would limit or control the biases.Third, the cross-sectional design limited the study of the longitudinal effects. Thus, a study on the efficacy of the SLT control policies with the changing regulations and taxes may be conducted longitudinally on a controlled trial of the same subjects. Fourth, the researchers evaluated the socio-contextual effects of SLT use, only on current users. Hence, the researchers excluded non-users and former users from the study. Future studies may investigate the role of socio-contextual factors by comparing the sample of both SLT users and non-users to understand the role of the factors in causing the distinction between the two behaviours. Finally, the study was conducted only on blue-collar workers. It will be worthwhile to study the behaviour of SLT use among the different levels of occupational status to identify the role of intellectual stress on SLT use at the worksite.

## Supplementary Information

Below is the link to the electronic supplementary material.

Supplementary Material 1

## Data Availability

The datasets generated and/or analyzed during the current study are available from the author on reasonable request. Please contact Dr. Anupam Bandyopadhyay at anupambandyopadhyay17@gmail.com.
