# Supplementary material for: Unveiling the socio contextual triggers of smokeless tobacco use among blue collar workers: implications for workplace health and policy
Source: Sci Rep. 2025 Nov 18;15:40544. doi: 10.1038/s41598-025-24275-2 (PMC12627588; doi:10.1038/s41598-025-24275-2)
Supplement: Supplementary file 1 — Supplementary Material 1 [file 41598_2025_24275_MOESM1_ESM.docx]

**supp. table 1**

**The Fagerström Test for Nicotine Dependence-Smokeless Tobacco (FTND-ST)-Modified**

| **Items** | **Options/Responses** | **Points** |
| --- | --- | --- |
| How soon after you wake up to do you place your first dip? | Within 5 minutes  6 – 30 minutes  31 – 60 minutes  After 60 minutes | 3  2  1  0 |
| How often do you intentionally swallow tobacco juice? | Always  Sometimes  Never | 2  1  0 |
| Which chew would you hate to give up most? | The first one in the morning  Any other | 1  0 |
| Original item :  How many cans/pouches per week do you use?  Modified item:  On an average how many times do you take SLT/day? | More than 3  2-3  1  More than 15  10 – 15  1- 9 | 2  1  0  2  1  0 |
| Do you chew more frequently during the first hours after awakening than during the rest of the day? | Yes  No | 1  0 |
| Do you chew if you are so ill that you are in bed most of the day? | Yes  No | 1  0 |

**supp. table 2**

**Latent constructs, observed items and their source of adoption**

| **Constructs** | **Instrument Items** | Reference |
| --- | --- | --- |
| Personality (PR) | My mood often goes up and down  I am a talkative person  I would be worried if I had a debt  I am a lively person  I prefer to go my own way rather than act by the rules  I am a nervous person  I worry a lot when there is any problem  I usually keep quiet on social occasions.  I am worried if I know there are mistakes in my work.  I am mostly quiet when I am with other people  I often feel lonely  It is better to follow society’s rules than go your own way | Eyesenck Personality Questionnaire- Revised short form (EPOR-S)  (Eyesenck and Eyesenck, 1992);  (Tiwari, Singh & Singh, 2009) |
| Neighbourhood Disadvantage (ND) | I trust most people in my local area  My local area has reputation as a safe place  In my local area neighbors look after each other  I am better off in my local area compared to others | Siapush et al., 2006 |
| Social Norm & Support (SNS) | Most of the people in my society use SLT  Wealthy people use SLT more  It is better to use SLT than to smoke or drink alcohol  Visitors freely use SLT in my home  My SLT use has increased due to the practice at my workplace  Many of my close friends and family members use SLT  SLT is more used when I am with others in a group  I started using SLT after seeing my parents using it at home  Sharing SLT helps in making friends with people | Adkinson et al., 2015  ITC-TCP- India (wave 2)  Biener et al, 2010  Stuber, Galea & Link, 2008  Biener et al, 2010 |
| Knowledge about the health effects of SLT use (KHE) | I know using SLT cause cancer (mouth, throat, etc.)  I often think about the harm SLT use might be doing to me  Nicotine is the main substance in smokeless tobacco that makes people use it  I know using SLT cause diseases of heart | ITC-TCP- India (wave 2) |
| Working Conditions (WC) | I have to fully focus my attention during work  I have to do/decide things where mistakes could be quite costly  I have to talk with peers, supervisor and other staffs for my work  My work is physically difficult  My job interferes with my family life  The physical surroundings of my workplace are pleasant  I can usually decide when to work fast and when to take it easy  My opinion is asked when decisions are made about my own jobs or about general problems in the work place  My work helps me to learn and develop new and special skills  The work I do is very interesting | Kawada & Otsuka, 2010  Albertsen et al., 2003; House et al, 1979  Hu & Cheng, 2010  House et al, 1979; Albersten et al, 2003  House et al, 1979  House et al, 1979  House et al, 1979; Kawada & Otsuka, 2010  Kawada & Otsuka, 2010  Hu & Cheng, 2010  House et al, 1979 |
| Perceived Effectiveness of SLT control Policies (PEP) | Anti-SLT advertising is effective for diminishing its use  There should be strong anti-SLT policies at the workplace  The pictorial warnings on SLT packages are realistic  The anti- tobacco advertising on television and movie theaters has made me more likely to quit using SLT | Raut & Pawar, 2016  ITC-TCP- India (wave 2) |
